# Intrathecal cytokine profile in neuropathy with anti‐neurofascin 155 antibody

**DOI:** 10.1002/acn3.50931

**Published:** 2019-10-27

**Authors:** Hidenori Ogata, Xu Zhang, Ryo Yamasaki, Takayuki Fujii, Akira Machida, Nobutoshi Morimoto, Kenichi Kaida, Teruaki Masuda, Yukio Ando, Motoi Kuwahara, Susumu Kusunoki, Yuri Nakamura, Takuya Matsushita, Noriko Isobe, Jun‐ichi Kira

**Affiliations:** ^1^ Department of Neurology Neurological Institute Graduate School of Medical Sciences Kyushu University Fukuoka Japan; ^2^ Department of Neurology and Tianjin Neurological Institute Tianjin Medical University General Hospital Tianjin China; ^3^ Department of Neurology Tsuchiura Kyodo General Hospital Ibaraki Japan; ^4^ Department of Neurology Kagawa Prefectural Central Hospital Kagawa Japan; ^5^ Department of Neurology Anti‐aging and Vascular Medicine National Defense Medical College Saitama Japan; ^6^ Department of Neurology Graduate School of Medical Sciences Kumamoto University Kumamoto Japan; ^7^ Department of Neurology School of Medicine Kinki University Osaka Japan; ^8^ Department of Neurological Therapeutics Neurological Institute Graduate School of Medical Sciences Kyushu University Fukuoka Japan

## Abstract

**Objective:**

To characterize the CSF cytokine profile in chronic inflammatory demyelinating polyneuropathy (CIDP) patients with IgG4 anti‐neurofascin 155 (NF155) antibodies (NF155^+^ CIDP) or those lacking anti‐NF155 antibodies (NF155^−^ CIDP).

**Methods:**

Twenty‐eight CSF cytokines/chemokines/growth factors were measured by multiplexed fluorescent immunoassay in 35 patients with NF155^+^ CIDP, 36 with NF155^−^ CIDP, and 28 with non‐inflammatory neurological disease (NIND).

**Results:**

CSF CXCL8/IL‐8, IL‐13, TNF‐α, CCL11/eotaxin, CCL2/MCP‐1, and IFN‐γ were significantly higher, while IL‐1β, IL‐1ra, and G‐CSF were lower, in NF155^+^ CIDP than in NIND. Compared with NF155^−^ CIDP, CXCL8/IL‐8 and IL‐13 were significantly higher, and IL‐1β, IL‐1ra, and IL‐6 were lower, in NF155^+^ CIDP. CXCL8/IL‐8, IL‐13, CCL11/eotaxin, CXCL10/IP‐10, CCL3/MIP‐1α, CCL4/MIP‐1β, and TNF‐α levels were positively correlated with markedly elevated CSF protein, while IL‐13, CCL11/eotaxin, and IL‐17 levels were positively correlated with increased CSF cell counts. IL‐13, CXCL8/IL‐8, CCL4/MIP‐1β, CCL3/MIP‐1α, and CCL5/RANTES were decreased by combined immunotherapies in nine NF155^+^ CIDP patients examined longitudinally. By contrast, NF155^−^ CIDP had significantly increased IFN‐γ compared with NIND, and exhibited positive correlations of IFN‐γ, CXCL10/IP‐10, and CXCL8/IL‐8 with CSF protein. Canonical discriminant analysis of cytokines/chemokines revealed that NF155^+^ and NF155^−^ CIDP were separable, and that IL‐4, IL‐10, and IL‐13 were the three most significant discriminators.

**Interpretation:**

Intrathecal upregulation of type 2 helper T (Th2) cell cytokines is characteristic of IgG4 NF155^+^ CIDP, while type 1 helper T cell cytokines are increased in CIDP regardless of the presence or absence of anti‐NF155 antibodies, suggesting that overproduction of Th2 cell cytokines is unique to NF155^+^ CIDP.

## Introduction

Chronic inflammatory demyelinating polyneuropathy (CIDP) is an acquired immune‐mediated disease involving the peripheral nerves. Both cell‐mediated and humoral immunity are thought to play pathogenic roles in CIDP.^1^ However, the precise mechanisms of CIDP remain to be elucidated, mainly because CIDP encompasses etiologically heterogeneous conditions. Recently, subsets of CIDP patients were reported to harbor autoantibodies against paranodal proteins, such as neurofascin 155 (NF155),^2^, ^3^, ^4^, ^5^ contactin‐1 (CNTN1),^6^, ^7^ and contactin‐associated protein 1 (CASPR1).^8^ Each of these autoantibodies is associated with unique features.^2^, ^3^, ^4^, ^5^, ^6^, ^7^, ^8^ However, it remains unclear why each paranodal autoantibody produces a specific manifestation, given that they bind to the same paranodal complex.

Anti‐NF155 antibodies found in a fraction of CIDP patients mainly belong to the immunoglobulin G (IgG)4 subclass.^3^ IgG4 anti‐NF155 antibody‐positive CIDP (NF155^+^ CIDP) demonstrates distinctive features, including younger age at onset, higher frequencies of drop foot, sensory ataxia, and tremor, marked prolongation of distal and F wave latency, extremely high cerebrospinal fluid (CSF) protein amounts, and marked hypertrophy of nerve roots on magnetic resonance neurography.^2^, ^3^, ^5^ However, biopsied sural nerves from IgG4 NF155^+^ CIDP patients lack inflammation and onion bulb formation, with only subperineurial edema and minimal paranodal demyelination.^3^ By electron microscopy, detachment of terminal myelin loops is characteristic for NF155^+^ CIDP, but not for anti‐NF155 antibody‐negative CIDP (NF155^−^ CIDP).^9^, ^10^

IgG4 cannot activate complement because it does not bind C1q.^11^ *In vivo*, IgG4 is monovalent and bispecific because of half molecular exchange, and does not internalize target antigens.^11^ Therefore, IgG4 autoantibodies only block protein–protein interactions,^11^ which explains why sural nerve pathology only presents paranodal terminal loop detachment in the absence of inflammation.^9^, ^10^ Restoration of nerve conduction by plasma exchange with decreased anti‐NF155 antibody titers is compatible with the blocking antibody action of IgG4.^12^ By contrast, extensive proximal nerve hypertrophy and pronounced CSF protein elevation, suggesting severe inflammation and/or edema of nerve roots, is unique to this condition,^3^ but is difficult to explain solely by IgG4 antibody functions. Recently, a strong association of certain *human leukocyte antigen* (*HLA*) *class II* alleles with NF155^+^ CIDP was reported in a European series,^13^ suggesting HLA class II‐restricted T‐cell involvement. These observations prompted us to clarify the CSF cytokine profile in patients with IgG4 NF155^+^ CIDP to elucidate the mechanism.

## Subjects and Methods

### Subjects

Thirty‐five consecutive IgG4 NF155^+^ CIDP and 36 NF155^−^ CIDP patients were enrolled in the present study. None of the NF155^+^ or NF155^−^ CIDP patients had anti‐NF186 or anti‐CNTN1 antibodies in sera. Among these patients, 44 were thoroughly examined in the Department of Neurology at Kyushu University Hospital between 01 January 2001 and 31 May 2018, while the other patients were referred to our department for an anti‐NF155 antibody assay between 1 November 2014 and 31 March 2018. All CIDP patients fulfilled the definite electrodiagnostic criteria of the European Federation of Neurological Societies/Peripheral Nerve Society for the diagnosis of CIDP,^14^ except for one NF155^+^ CIDP patient who showed no evoked potentials on nerve conduction studies. Clinical features of 11 NF155^+^ CIDP patients and biopsied sural nerve pathologies of 3 NF155^+^ CIDP patients showing subperineurial edema without inflammatory cell infiltrates were previously reported elsewhere.^3^, ^10^ Hughes functional grading^15^ was used to evaluate clinical severity. Twenty‐two IgG4 NF155^+^ CIDP and 23 NF155^−^ CIDP patients had received no treatment at the time of lumbar puncture (LP). Furthermore, two or more CSF samples at different time points were available in nine NF155^+^ CIDP patients. For controls, 28 other non‐inflammatory neurological disease (NIND) patients were enrolled, including 12 with amyotrophic lateral sclerosis, five with spondylosis, four with normal pressure hydrocephalus, three with spinocerebellar ataxia, and one each with metabolic neuropathy, hyperthyroidism, psychosomatic disorder, or cerebral venous malformations. The research protocols for the study were approved by the Kyushu University Ethics Committee. An opt‐out recruitment method was adopted.

### Sample collection

CSF samples were obtained by non‐traumatic LP. None of the subjects for CSF analysis had any ongoing recent infection at the time of LP. The periods (disease durations) from onset of symptoms to CSF sample collection were comparable between NF155^+^ and NF155^−^ CIDP patients (Table 1). CSF samples were immediately centrifuged at 800 rpm (100*g*) at 4°C for 5 min, and the supernatants were stored at −80°C until analysis.

**Table 1:** Demographic characteristics of the CIDP and NIND patients.

	NF155^+^ (*n* = 35)	NF155^−^ (*n* = 36)	NIND (*n* = 28)	*P‐*value*		
				NF155^+^ *vs.* NF155^−^	NF155^+^ *vs.* NIND	NF155^−^ *vs.* NIND
Female:Male	1:2.5 (28.6%)	1:2.3 (30.6%)	1:1.8 (35.7%)	NS	NS	NS
Age at sample collection, median (range), y	30 (14–66)	56.5 (11–81)	61.5 (32−81)	<0.0001	<0.0001	NS
Age at onset, median (range), y	25 (13–64)	46 (10–76)	NA	0.0001	NA	NA
Disease duration at sample collection, median (range), m	9 (3–456)	14 (0–414)	NA	NS	NA	NA
Hughes functional grade at sample collection, median (range)	2 (1–4)	NA	NA	NA	NA	NA
Therapeutic status at sample collection	n/N (%)	n/N (%)	n/N (%)			
Without treatment	22/35 (62.9%)	23/36 (63.9%)	NA	NS	NA	NA
With treatment Type of treatment	13/35 (37.1%)	13/36 (36.1%)	NA	NS	NA	NA
Plasma exchange	2/13 (15.4%)	3/13 (23.1%)	NA	NS	NA	NA
Steroid pulse	11/13 (84.6%)	6/13 (46.2%)	NA	NS	NA	NA
Oral steroid	10/13 (76.9%)	8/13 (61.5%)	NA	NS	NA	NA
IVIg	12/13 (92.3%)	9/13 (69.2%)	NA	NS	NA	NA
Other immunotherapies	2/13 (15.4%)	6/13 (46.2%)	NA	NS	NA	NA
Clinical phenotype	n/N (%)	n/N (%)	n/N (%)			
Typical	20/35 (57.1%)	24/36 (66.7%)	NA	NS	NA	NA
DADS	13/35 (37.1%)	2/36 (5.6%)	NA	0.0013	NA	NA
MADSAM	1/35 (2.9%)	5/36 (13.9%)	NA	NS	NA	NA
Others	1/35 (2.9%)	5/36 (13.9%)	NA	NS	NA	NA

CIDP, chronic inflammatory demyelinating polyneuropathy; DADS, distal acquired demyelinating symmetric neuropathy; IVIg, intravenous immunoglobulin; m, month; MADSAM, multifocal acquired demyelinating sensory and motor neuropathy; NA, not applicable; NF155, neurofascin 155; NF155^+^, immunoglobulin G4 anti‐neurofascin 155 antibody‐positive CIDP; NF155^−^, anti‐NF155 antibody‐negative CIDP; n/N, positive patient number/ total examined patient number; NS, not significant; NIND, other non‐inflammatory neurological disease; y, year.

* *P* < 0.05 = significant difference.

### Anti‐NF155, anti‐NF186, and anti‐CNTN1 antibodies and IgG subclass analysis

IgG and IgG4 anti‐NF155 antibodies as well as anti‐NF186 and anti‐CNTN1 antibodies in sera were measured by flow cytometry using human embryonic kidney 293 cell lines stably expressing human NF155, NF186, or CNTN1 as described previously.^3^, ^7^

### Multiplexed fluorescent immunoassay

The concentrations of the following 28 cytokines/chemokines/growth factors (Table S1) in the liquid phase of CSF were measured by multiplexed fluorescent bead‐based immunoassay as described previously:^16^, ^17^, ^18^, ^19^ pleiotropic cytokines, interleukin (IL)‐1β, IL‐6, tumor necrosis factor (TNF)‐α, and IL‐7; type 1 helper T cell (Th1)‐related cytokines, IL‐2, IL‐12 (p70), IL‐15, and interferon (IFN)‐γ; type 2 helper T cell (Th2)‐related cytokines, IL‐4, IL‐5, IL‐9, IL‐10, and IL‐13; type 17 helper T cell (Th17)‐related cytokine, IL‐17; follicular helper T (Tfh)‐related cytokine, IL‐21; chemokines, C‐X‐C motif ligand (CXCL)8/IL‐8, CCL11/eotaxin, CXCL10/interferon gamma‐inducible protein (IP)‐10, CCL2/monocyte chemoattractant protein (MCP)‐1, CCL3/macrophage inflammatory protein (MIP)‐1α, CCL4/MIP‐1β, and CCL5/regulated upon activation, normal T cell expressed and secreted (RANTES); growth factors, granulocyte colony‐stimulating factor (G‐CSF), granulocyte‐macrophage colony‐stimulating factor (GM‐CSF), platelet‐derived growth factor (PDGF)‐BB, basic fibroblast growth factor (bFGF), and vascular endothelial growth factor (VEGF); and anti‐inflammatory cytokine, IL‐1 receptor antagonist (IL‐1ra). The Bio‐plex cytokine assay system (Bio‐Rad, Hercules, CA) was used according to the manufacturer’s instructions. The same lots of reagents were used throughout the experiments, and the interassay and intraassay variabilities were reported to be <10%.^16^, ^17^, ^18^, ^19^ All samples were diluted fourfold and analyzed in duplicate. Cytokine/chemokine/growth factor concentrations were calculated by reference to a standard curve for each molecule derived using various concentrations of the standard assayed in the same manner as the CSF samples. The detection limit for each molecule was determined by the recovery of the corresponding standard and the lowest value with >70% recovery was set as the lower detection limit.^16^, ^17^, ^18^, ^19^ No samples were beyond the upper detection limits, while some samples were below the lower detection limits. Each cytokine/chemokine and growth factor showed different detection rates (Table S1).

### Statistical analysis

Cytokines/chemokines/growth factors with detection rates <30% were excluded from analysis as previously reported.^16^, ^17^, ^18^, ^19^ The chi‐square test was performed to evaluate the statistical significance of detection rates of cytokines/chemokines/growth factors among NF155^+^ CIDP, NF155^−^ CIDP, and NIND patients. If the *P* < 0.05, Fisher’s exact probability test was used for comparisons between any two groups, followed by Bonferroni–Dunn’s correction. The Kruskal–Wallis test was performed to compare the levels of cytokines/chemokines/growth factors between three groups. If the *P* < 0.05, the Mann–Whitney *U*‐test was used for comparisons between any two groups, followed by Bonferroni–Dunn’s correction. The Wilcoxon signed‐rank test was performed to evaluate the longitudinal analysis according to treatment status. Spearman’s rank correlation coefficient was used for statistical analyses between cytokines/chemokines/growth factors and CSF protein amounts or cell counts. All of the above statistical analyses were performed with GraphPad Prism ver. 5.0 software (GraphPad, San Diego, CA). To separately analyze the correlations of CSF cytokines/chemokines in patients with NF155^+^ CIDP and NF155^−^ CIDP, heatmaps were generated by the “gplots” package in R on the basis of Spearman’s rank correlation coefficient and cluster analysis. A canonical discriminant analysis (CDA) was performed by JMP Pro software (ver. 13.0.0; SAS Institute, Cary, NC).

## Results

### Clinical findings

**Figure 1:**
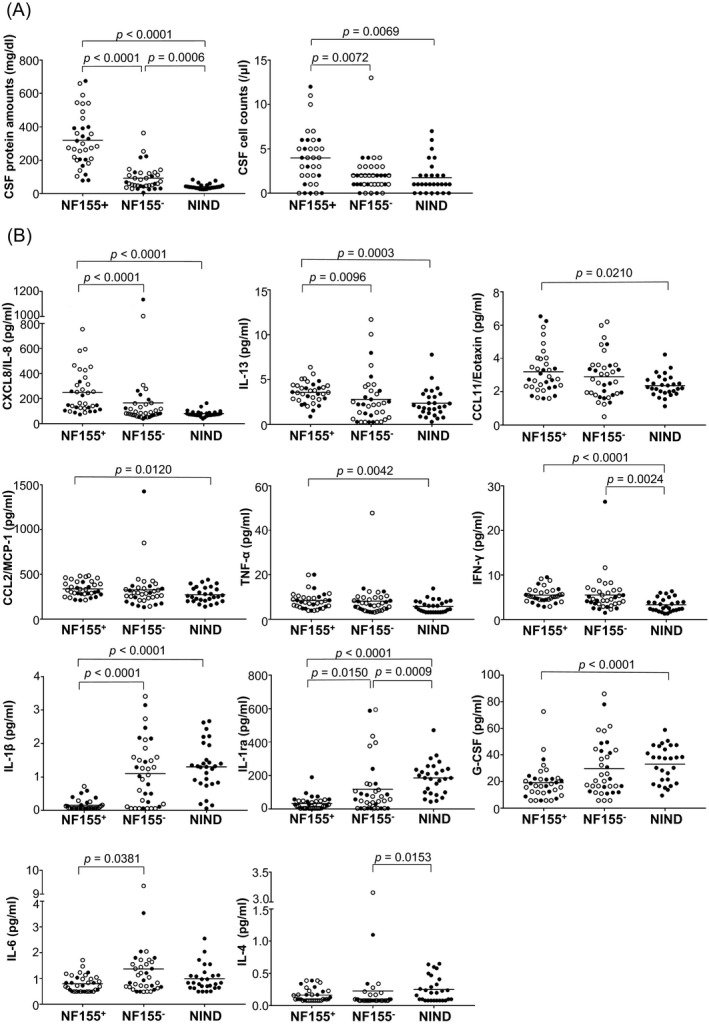
CSF protein amounts, cell counts, and cytokine/chemokine/growth factor levels. (A) CSF protein amounts and cell counts from all enrolled subjects. (B) Levels of CSF cytokines/chemokines and growth factors showing significant differences between immunoglobulin G (IgG)4 anti‐neurofascin 155 (NF155) antibody‐positive CIDP (NF155^+^), anti‐NF155 antibody‐negative CIDP (NF155^−^), and other non‐inflammatory neurological disease (NIND) patients. Samples from untreated patients are indicated by open circles. The cytokines that showed significant changes are indicated by p values while other cytokines did not show any significant changes when compared among groups. CCL = C‐C motif ligand; CIDP = chronic inflammatory demyelinating polyneuropathy; CSF = cerebrospinal fluid; CXCL = C‐X‐C motif ligand; G‐CSF = granulocyte‐colony stimulating factor; IFN = interferon; IL = interleukin; IL‐1ra = interleukin‐1 receptor antagonist; MCP‐1 = monocyte chemoattractant protein‐1; TNF‐α = tumor necrosis factor‐α.

The demographic features of the 35 IgG4 NF155^+^ CIDP, 36 NF155^−^ CIDP, and 28 NIND patients are summarized in Table 1. Age at onset was younger in NF155^+^ CIDP patients than in NF155^−^ CIDP patients (*P* = 0.0001). NF155^+^ CIDP patients showed a higher frequency of distal acquired demyelinating symmetric neuropathy (DADS) than NF155^−^ CIDP patients (*P = *0.0013), although more than half of NF155^+^ CIDP patients (57.1%) had typical CIDP. CSF protein amounts and cell counts were higher in NF155^+^ CIDP patients than in NF155^−^ CIDP and NIND patients (protein amounts, *P* < 0.0001 for both; cell counts, *P = *0.0072 and *P = *0.0069, respectively) (Fig. 1A). CSF protein amounts were also higher in NF155^−^ CIDP patients than in NIND patients (*P = *0.0006).

### Concentrations of CSF cytokines/chemokines/growth factors

The detection rates of cytokines/chemokines/growth factors in CSF are shown in Table S1. IL‐2, IL‐12, IL‐15, IL‐21, bFGF, GM‐CSF, and VEGF were excluded from further statistical analysis because of their low detection rates. NF155^+^ CIDP patients had higher levels of CXCL8/IL‐8 (*P* < 0.0001), IL‐13 (*P* < 0.0003), CCL11/eotaxin (*P* = 0.0210), CCL2/MCP‐1 (*P* = 0.0120), TNF‐α (*P* = 0.0042), and IFN‐γ (*P* < 0.0001) compared with NIND patients (Fig. 1B). CXCL8/IL‐8 and IL‐13 levels were even significantly higher in NF155^+^ CIDP patients compared with NF155^−^ CIDP patients (*P* < 0.0001 and *P* = 0.0096, respectively). IFN‐γ levels were also increased in NF155^−^ CIDP patients compared with NIND patients (*P* = 0.0024). By contrast, NF155^+^ CIDP patients had lower levels of IL‐1β and IL‐1ra than NF155^−^ CIDP and NIND patients (IL‐1β, *P* < 0.0001 for both; IL‐1ra, *P* = 0.0150 and *P* < 0.0001, respectively). NF155^+^ CIDP patients had lower G‐CSF levels than NIND patients (*P* < 0.0001) and lower IL‐6 levels than NF155^−^ CIDP patients (*P* = 0.0291). IL‐1ra and IL‐4 levels were lower in NF155^−^ CIDP patients than in NIND patients (*P* = 0.0009 and *P* = 0.0153, respectively). Even when only the pretreatment samples were evaluated, similar results were found (Fig. S1).

### Correlations of CSF cytokine/chemokine/growth factor levels with clinical parameters

**Figure 2:**
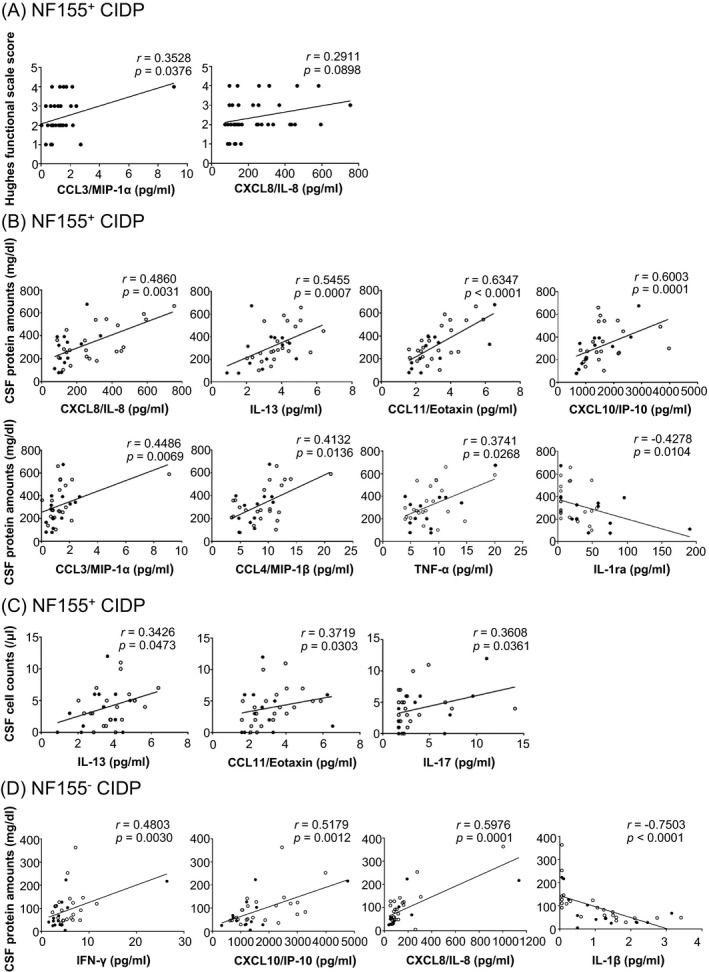
Correlations of cytokine/chemokine levels with clinical severity and protein amounts and cell counts in CSF. (A) Correlations between CSF cytokine/chemokine levels and Hughes functional scale grade scores in NF155^+^ CIDP. When the outliner showing the extremely high CCL3/MIP‐1α level was excluded, the CCL3/MIP‐1α levels still showed a tendency to be positively associated with Hughes functional grade scores (*r* = 0.2984, *P* = 0.0865). (B, C) CSF cytokines and chemokines showing correlations with CSF protein amounts (B) and cell counts (C) in NF155^+^ CIDP patients. (D) CSF cytokines and chemokines showing correlations with CSF protein amounts in NF155^−^ CIDP patients. Even when the two outliers showing the highest and second‐highest CXCL8/IL‐8 levels were excluded, the correlation remained statistically significant (*r* = 0.5216, *P* < 0.0016). Samples from untreated patients are indicated by open circles. Spearman’s rank correlation coefficient was used for this analysis. CCL = C‐C motif ligand; CIDP = chronic inflammatory demyelinating polyneuropathy; CSF = cerebrospinal fluid; CXCL = C‐X‐C motif ligand; G‐CSF = granulocyte colony‐stimulating factor; IFN = interferon; IL = interleukin; IP‐10 = interferon‐γ gamma‐inducible protein‐10; MIP = macrophage inflammatory protein; NF155 = neurofascin 155; NF155^+^ = IgG4 anti‐NF155 antibody‐positive; NF155^−^ = anti‐NF155 antibody‐negative; TNF‐α = tumor necrosis factor‐α.

When the correlations of CSF cytokine/chemokine/growth factor levels with clinical severity were examined in NF155^+^ CIDP patients, only CCL3/MIP‐1α levels showed a weak positive correlation with Hughes functional grade scores (*r* = 0.3528, *P* = 0.0376), although CXCL8/IL‐8 levels tended toward a positive correlation with the scores (*r* = 0.2911, *P* = 0.0898) (Fig. 2A). Levels of CXCL8/IL‐8, IL‐13, CCL11/eotaxin, CXCL10/IP‐10, CCL3/MIP‐1α, CCL4/MIP‐1β, and TNF‐α showed positive correlations with CSF protein concentrations in NF155^+^ CIDP patients (CXCL8/IL‐8, *r* = 0.4860, *P* = 0.031; IL‐13, *r* = 0.5455, *P* = 0.0007; CCL11/eotaxin, *r* = 0.6347, *P* < 0.0001; CXCL10/IP‐10, *r* = 0.6003, *P* = 0.0001; CCL3/MIP‐1α, *r* = 0.4486, *P* = 0.0069; CCL4/MIP‐1β, *r* = 0.4132, *P* = 0.0136; TNF‐α, *r* = 0.3741, *P* = 0.0268) (Fig. 2B), while IL‐1ra showed a negative correlation with CSF protein amounts (*r* = −0.4278, *P* < 0.0104). Levels of IL‐13, CCL11/eotaxin, and IL‐17 also demonstrated positive correlations with CSF cell counts in NF155^+^ CIDP (IL‐13, *r* = 0.3426, *P* = 0.0473; CCL11/eotaxin, *r* = 0.3719, *P* = 0.0303, IL‐17, *r* = 0.3608, *P* < 0.0361) (Fig. 2C). In NF155^−^ CIDP, IFN‐γ, CXCL10/IP‐10, and CXCL8/IL‐8 levels showed positive correlations with CSF protein amounts (IFN‐γ, *r* = 0.4803, *P* = 0.0030; CXCL10/IP‐10, *r* = 0.5179, *P* = 0.0012; CXCL8/IL‐8, *r* = 0.5976, *P* = 0.0001) (Fig. 2D) and IL‐1β showed a negative correlation with CSF protein amounts (*r* = −0.7503, *P* < 0.0001), while the CXCL10/IP‐10, CXCL8/IL‐8, and IL‐1β levels did not differ between NF155^−^ CIDP and NIND. Even when only the pretreatment samples were used, similar results were obtained (Fig. S2).

### Effects of immunotherapies on CSF cytokine/chemokine/growth factor levels

**Figure 3:**
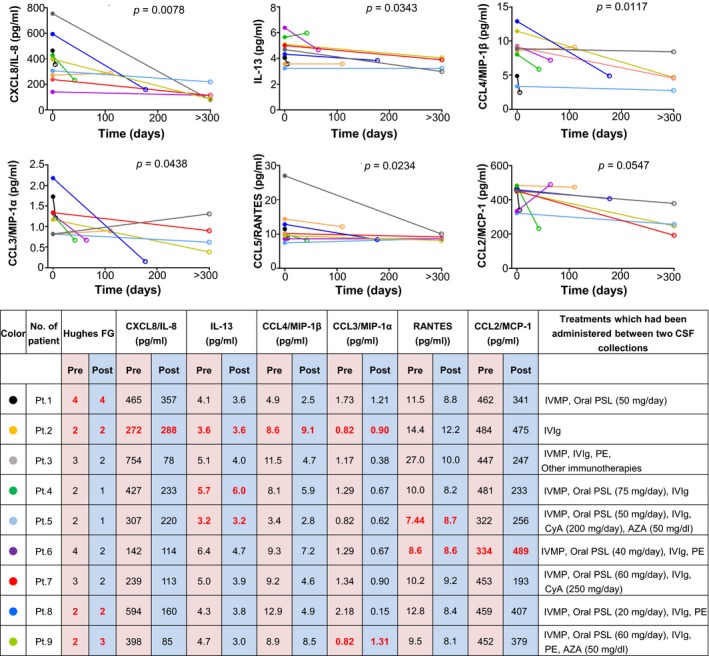
Changes in cytokine/chemokine levels after treatment in identical patients with IgG4 anti‐NF155 antibody‐positive CIDP. The Hughes FG system was used to evaluate clinical status (grade 0: normal; grade 1: minimal symptoms and signs, able to run; grade 2: able to walk 5 m independently; grade 3: able to walk 5 m with use of aids; grade 4: wheelchair user or bedbound; grade 5: requires assisted ventilation; grade 6: dead). Maximum doses of oral immunosuppressants are shown in parentheses. Unchanged or increased values in Hughes FG and cytokine/chemokine levels after treatment are indicated by bold red characters. AZA = azathioprine; CCL = C‐C motif ligand; CIDP = chronic inflammatory demyelinating polyneuropathy; CSF = cerebrospinal fluid; CyA = cyclosporine A; CXCL = C‐X‐C motif ligand; G‐CSF = granulocyte colony‐stimulating factor; Hughes FG = Hughes functional grade; IFN = interferon; IL = interleukin; IVIg = intravenous immunoglobulin; IVMP = intravenous methylprednisolone pulse therapy (1,000 mg/day for 3 consecutive days); MCP‐1 = monocyte chemoattractant protein‐1; PE = plasma exchange; PSL = prednisolone.

Longitudinal analyses of CSF cytokine/chemokine/growth levels in 9 patients with CSF samples repeatedly obtained before and after immunotherapy revealed that CXCL8/IL‐8, IL‐13, CCL4/MIP‐1β, CCL3/MIP‐1α, and CCL5/RANTES were decreased after treatment (*P = *0.0078, *P = *0.0343, *P = *0.0117, *P = *0.0438, and *P* = 0.0234, respectively) (Fig. 3). A similar tendency was observed for CCL2/MCP‐1 (*P = *0.0547). Five patients (Patients 3–7 in Fig. 3) treated with combined immunotherapies such as intravenous immunoglobulin (IVIg), corticosteroids, plasma exchanges, and immunosuppressants, showed improvement in Hughes functional grade scores together with deceases in most of these cytokines. By contrast, a patient treated with IVIg alone (Patient 2 in Fig. 3) showed increases in some of these cytokines and no clinical improvement. Subsequently, CSF cytokine/chemokine/growth factor levels were compared between 22 untreated and 13 treated samples from 35 NF155^+^ CIDP patients and between 23 untreated and 13 treated samples from 36 NF155^−^ CIDP patients, in which one treated or untreated sample was evaluated per patient. CXCL8/IL‐8 and CCL2/MCP‐1 levels were lower and IL‐1ra levels were higher in treated samples than in untreated samples from NF155^+^ CIDP patients (*P = *0.0043, *P = *0.0077, and *P = *0.0241, respectively) (Fig. S3A). In NF155^−^ CIDP, IFN‐γ levels were significantly lower in treated samples than in untreated samples (*P* = 0.0442) (Fig. S3B).

### Cluster analyses of CSF cytokines/chemokines/growth factors

**Figure 4:**
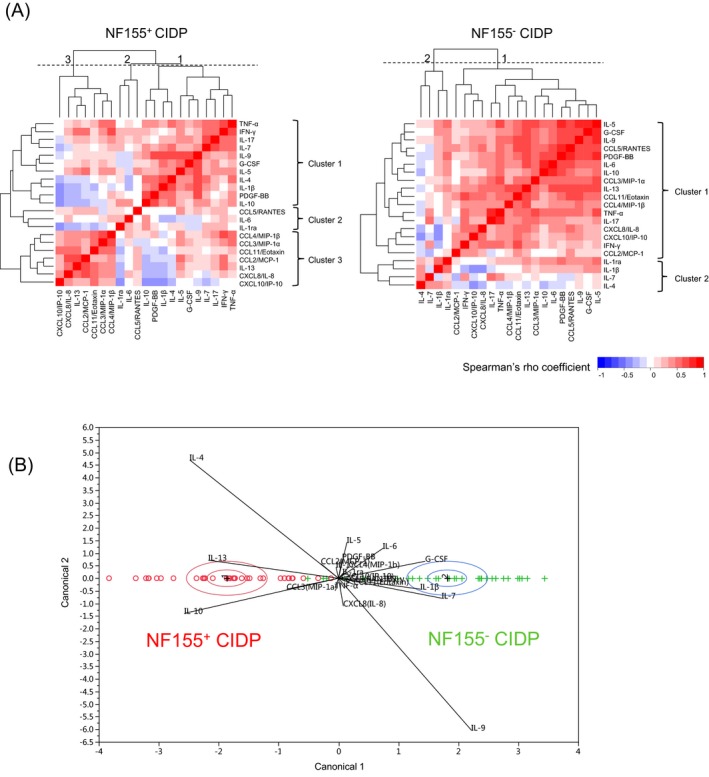
Cluster analysis of CSF cytokines/chemokines/growth factors in IgG4 anti‐NF155 antibody‐positive and ‐negative CIDP. (A) Clustering of correlations between each CSF cytokine level in patients with NF155^+^ (left panel) and NF155^−^ (right panel) CIDP, respectively. Among the cytokines/chemokines/growth factors analyzed, the distance of each pair of cytokines/chemokines/growth factors based on Spearman’s correlation coefficient is shown as a heatmap. In NF155^+^ CIDP patients, there were three major clusters (clusters 1, 2, and 3). In NF155^−^ CIDP patients, there were two major clusters (clusters 1 and 2). The numbers on each heatmap (1, 2, and 3) indicate the cut‐off position for each cluster. Dotted line: cut‐off level. (B) Canonical plot for 35 NF155^+^ (red) and 36 NF155^−^ (green) CIDP patients. CIDP = chronic inflammatory demyelinating polyneuropathy; CSF = cerebrospinal fluid; CCL = C‐C motif ligand; CXCL = C‐X‐C motif ligand; G‐CSF: granulocyte colony‐stimulating factor; IFN = interferon; IL = interleukin; IP‐10 = interferon‐γ‐inducible protein‐10; MCP‐1 = monocyte chemoattractant protein‐1; NF155 = neurofascin 155; NF155^+^ = IgG4 anti‐NF155 antibody‐positive CIDP; NF155^−^ = anti‐NF155 antibody‐negative CIDP; PDGF = platelet‐derived growth factor; RANTES = regulated upon activation, normal T cell expressed and secreted.

Cluster analyses in NF155^+^ CIDP showed three major clusters (Fig. 4A). Cluster 1 comprised TNF‐α, IFN‐γ, IL‐17, IL‐7, IL‐9, G‐CSF, IL‐5, IL‐4, IL‐1β, PDGF‐BB, and IL‐10, cluster 2 consisted of CCL5/RANTES, IL‐6, and IL‐1ra, and cluster 3 contained CCL4/MIP‐1β, CCL3/MIP‐1α, CCL11/eotaxin, CCL2/MCP‐1, IL‐13, CXCL8/IL‐8, and CXCL10/IP‐10. Distances between the cytokines in cluster 2 were greater than those in the other clusters, suggesting that this cluster was less meaningful than the other two clusters. By contrast, there were 2 clusters in NF155^−^ CIDP, which showed a different pattern of results from NF155^+^ CIDP. One large cluster comprised most of the cytokines/chemokines examined, with universal positive correlations among the cytokines, while the other cluster included IL‐1ra, IL‐1β, IL‐4, and IL‐7. Furthermore, CDA using the measured cytokine/chemokine/growth factor levels showed that NF155^+^ CIDP was clearly separated from NF155^−^ CIDP on the canonical 1 axis, wherein NF155^+^ CIDP was highly associated with IL‐13, IL‐4, and IL‐10 (Fig. 4B), suggesting that these cytokine levels are major discriminants between the two conditions.

### Subgroup analysis of NF155^−^ CIDP

Finally, we classified NF155^−^ CIDP patients into two subgroups by IL‐1β levels: those with median (1.06 mg/dl) or higher IL‐1β levels and those with lower than median IL‐1β levels. As a result, we found that the frequencies of typical CIDP and CSF protein levels were significantly higher in the low IL‐1β group than in the high IL‐1β group (*P* = 0.0063 and *P* < 0.0001, respectively) (Table S2). Sex ratio, CSF cell counts, and age at onset were comparable between the two subgroups.

## Discussion

In this study, the characteristic features of IgG4 NF155^+^ CIDP were marked elevations of Th2‐ and Th1‐related cytokines. By contrast, NF155^−^ CIDP showed a significant increase in IFN‐γ only and positive correlations of IFN‐γ and CXCL10/IP‐10 (chemokine downstream of IFN‐γ) with CSF protein amounts, suggesting a Th1 shift. Cluster analyses further supported distinct cytokine profiles between NF155^+^ and NF155^−^ CIDP. Upregulation of cluster 3 cytokines, including IL‐13, CCL11/eotaxin, CCL2/MCP‐1, CCL3/MIP‐1α, CCL4/MIP‐1β, CXCL8/IL‐8, and CXCL10/IP‐10, was characteristic of NF155^+^ CIDP. Thus, we consider that co‐upregulation of Th2 and Th1 cytokines is a unique feature of NF155^+^ CIDP. An intrathecal Th1 shift has been proposed in CIDP, based on elevated levels of Th1‐related cytokines including IL‐12 and CXCL10/IP10 together with increased IFN‐γ^+^IL‐4^−^CD4^+^ T (Th1) cell percentages in CSF cells,^20^ although no reports have described CIDP subtype‐specific CSF cytokine profiles. Because NF155^+^ CIDP comprises only a minority of total CIDP, the cytokine profile of total CIDP patients may have not reflected Th2 cytokine upregulation in NF155^+^ CIDP patients in previous studies.^20^, ^21^, ^22^, ^23^, ^24^

IgG4 usually arises after chronic exposure to antigenic stimuli, and its physiological role is to compete with IgE and block its pathogenic effects in allergic responses.^25^ The class switch to IgG4 is facilitated in the presence of Th2 cytokines including IL‐13, IL‐4, and IL‐10.^26^ Intriguingly, CDA revealed that IL‐13, IL‐4, and IL‐10 levels were the major discriminants between NF155^+^ and NF155^−^ CIDP, suggesting a key role for these Th2 cytokines in NF155^+^ CIDP. Furthermore, the positive correlations of Th2 cytokines (IL‐13 and CCL11/eotaxin) with CSF cell counts and protein amounts and reduction of IL‐13 levels during longitudinal follow‐up after immunotherapy in NF155^+^ CIDP suggested the involvement of Th2 cells in spinal root inflammation, in addition to the induction of IgG4 autoantibodies. In Th2 inflammation, IL‐13 is a major effector cytokine, while CCL11/eotaxin exhibits potent chemotactic activity.^27^, ^28^

The decreases in CXCL8/IL‐8, CCL2/MCP‐1, CCL3/MIP‐1α, and CCL4/MIP‐1β, as downstream chemokines stimulated by IL‐13,^29^, ^30^ after immunotherapy in parallel with some clinical improvement reflected in Hughes functional grade scores suggest a direct involvement of these chemokines in NF155^+^ CIDP. The tendency toward positive correlations of CCL3/MIP‐1α and CXCL8/IL‐8 with Hughes functional grade scores may support an involvement of these chemokines in nerve damage, although further prospective evaluation of neurological impairment is required to confirm the correlations between CSF cytokine levels and disability. CXCL8/IL‐8 is secreted by a variety of cells including blood monocytes, fibroblasts, endothelial cells, and epithelial cells upon stimulation by various cytokines, leading to 10–100‐fold upregulation of CXCL8/IL‐8 expression.^31^ As the changes in levels of downstream cytokines, such as CXCL8/IL‐8, are much more amplified than those of upstream cytokines, the reduction in CXCL8/IL‐8 levels by immunotherapy could be more prominent than those in IL‐13 and CCL11/eotaxin levels, suggesting that these downstream chemokines may be potential biomarkers that can reflect disease activity. In addition, IFN‐γ was significantly increased in NF155^+^ CIDP patients compared with NIND patients, also suggesting the potential involvement of Th1 cells. Because we found a small but significant increase in CSF cells in pretreated NF155^+^ CIDP patients, a more detailed analysis of CSF cytology, including helper T cells and eosinophils, is required to elucidate the effector mechanism of root inflammation.

IL‐1β and IL‐1ra were significantly decreased in NF155^+^ CIDP patients compared with NF155^−^ CIDP and NIND patients. Th2 cytokines such as IL‐13 were reported to decrease IL‐1β production,^32^ suggesting the suppression of these cytokines by activated Th2 cells in NF155^+^ CIDP. Macrophage‐mediated demyelination, which is often seen in biopsied sural nerves of NF155^−^ CIDP patients,^9^, ^33^ was not observed in those of NF155^+^ CIDP patients.^9^, ^33^ Because IL‐1β activates macrophages,^34^ the pronounced suppression of IL‐1β in NF155^+^ CIDP may be related to the absence of macrophage‐mediated demyelination. Even in NF155^−^ CIDP patients, the low IL‐β subgroup showed higher frequencies of typical CIDP (nearly 90%) and higher CSF protein levels than the high IL‐β subgroup. Thus, in the low IL‐β subgroup preferentially presenting typical CIDP, a mechanism other than macrophage‐mediated demyelination could be operative. By contrast, a macrophage‐mediated mechanism may be operative in the high IL‐β subgroup. Although a decrease in IL‐1ra was common in both NF155^+^ and NF155^−^ CIDP, the decrease was most marked in NF155^+^ CIDP. IL‐1ra is a representative anti‐inflammatory cytokine,^34^ and thus the pronounced decrease in IL‐1ra levels in NF155^+^ CIDP patients may also contribute to severe spinal root inflammation.

Biopsied sural nerves showed marked subperineurial edema, suggesting disruption of the blood–nerve barrier (BNB), although no onion bulb formation was observed.^3^, ^10^ Therefore, distal nerve edema may be a manifestation of widespread BNB breakdown including the nerve roots. However, it is difficult to explain the mechanism for how IgG4 anti‐NF155 antibodies targeting the paranodal structure without activating complement can induce disruption of the BNB. Because proinflammatory cytokines/chemokines, such as IL‐13, CCL‐11/eotaxin, and CXCL8/IL‐8, were increased in NF155^+^ CIDP patients including three biopsied cases and showed significant positive correlations with CSF protein levels in our study, these cytokines/chemokines and the immunocytes producing them may be directly or indirectly involved in BNB disruption.^35^, ^36^

Poor responsiveness of NF155^+^ CIDP to IVIg has repeatedly been reported by us and others.^2^, ^3^, ^4^ Plausible mechanisms of action for IVIg in inflammatory neuropathies include inhibition of the complement pathway, modulation of Fc receptors on macrophages, anti‐idiotype antibody production, inhibition of cell migration by modulation of adhesion molecules, and promotion of remyelination.^37^ Poor responses to IVIg in NF155^+^ CIDP may be explained by the lack of complement‐mediated inflammatory cascade and macrophage‐mediated demyelination in NF155^+^ CIDP, as shown by pathological studies of biopsied sural nerves.^9^, ^10^ This notion is also supported by the unique feature of IgG4, namely the inability to activate complement.^11^ Efficacy of B‐cell depletion therapy using rituximab, a chimeric anti‐CD20 antibody, on NF155^+^ CIDP was recently reported in a small case series.^38^, ^39^ B cells produce antibodies, present antigens to T cells together with costimulatory signals, and secrete proinflammatory cytokines.^40^ Although the exact mechanism for B‐cell depletion therapy in CIDP remains to be elucidated, attenuation of B‐T cell interactions may be partly responsible for the amelioration of disease activity given the marked elevations of CSF Th2 and Th1 cytokines in NF155^+^ CIDP. Indeed, combined immunotherapies, but not IVIg alone, decreased the concentrations of proinflammatory cytokines/chemokines, such as CXCL8/IL‐8, IL‐13, CCL3/MIP‐1α, and CCL4/MIP‐1β in CSF together with clinical improvement in our preliminary follow‐up study, suggesting that downregulation of intrathecal proinflammatory cytokines/chemokines is critical.

This study had some limitations. First, we did not measure anti‐CASPR1 antibodies in our patients. However, because only one CIDP case with anti‐CASPR1 antibodies has been reported in the literature,^8^ we believe that this issue would not severely distort the present results. Second, because IgG4 NF155^+^ CIDP is a rare disease, the present cases were collected from all over Japan. Thus, data and sample collection may not be uniform among the participating institutions, and only post‐treatment samples were available in some cases. Nevertheless, IgG4 NF155^+^ CIDP patients demonstrated relatively similar study results, and thus our preliminary CSF findings provide insights into its mechanism.

## Author Contributors

HO, XZ, and JK designed and conceptualized the study, collected the data, analyzed the data, and drafted the manuscript. RY, YN, and TM collected data, analyzed the data, and drafted the manuscript. TF, AK, NM, KK, TM, YA, MK, SK, NI contributed to data collection, fidelity checking, data cleaning, and data interpretation.

## Conflicts of Interest

HO received a grant from JSPS KAKENHI (Grant No. 18K15454) and received honoraria from Japan Blood Products Organization and CSL Behring. XZ received financial support from the China Scholarship Council. RY received a grant from JSPS KAKENHI (Grant No. 16K09694). MK received honoraria from Teijin, Nihon Pharmaceutical and Japan Blood Products Organization. SK received grants from Ministry of Health, Labour and Welfare of Japan, the Japan Agency for Medical Research and Development (AMED) and Ministry of Education, Culture, Sports, Science and Technology of Japan, and received consultant fees, speaking fees and/or honoraria from Teijin, Japan Blood Product Organization and Nihon Pharmaceutical. YN received a grant and salary from Mitsubishi Tanabe Pharma, Bayer Yakuhin, Ltd., and Japan Blood Products Organization. TM received speaker honoraria payments from Mitsubishi Tanabe Pharma, Takeda Pharmaceutical Company, and Biogen Japan. NI is supported by a grant from JSPS KAKENHI (Grant No. 18K07529), and received grant support from Mitsubishi Tanabe Pharma, Osoegawa Neurology Clinic, Bayer Yakuhin, Ltd., and Japan Blood Products Organization. JK is supported by grants from JSPS KAKENHI (Grant No. 16H02657), Health and Labour Sciences Research Grants on Intractable Diseases (H29‐Nanchitou (Nan)‐Ippan‐043), and grants from the Japan Agency for Medical Research and Development (AMED) under Grant Number JP17ek0109115 and JP18ek0109376, and received consultant fees, speaking fees and/or honoraria from Novartis Pharma, Mitsubishi Tanabe Pharma, Boehringer Ingelheim, Teijin Pharma, Takeda Pharmaceutical Company, Otsuka Pharmaceutical, Astellas Pharma, Pfizer Japan, and Eisai. TF, AM, NM, KK, TM and YA report no disclosures.

## Supporting information


**Table S1** **.** Detection rates of cytokines/chemokines/growth factors in CSF.
**Table S2** **.** Comparisons of clinical and laboratory findings between anti‐NF155 antibody‐negative CIDP patients with high and low IL‐1β levels.
**Figure S1** **.** CSF protein amounts, cell counts, and cytokine/chemokine/growth factor levels in untreated patients.
**Figure S2** **.** Correlations of cytokine/chemokine levels with clinical severity and CSF protein amounts and cell counts in untreated patients.
**Figure S3** **.** Comparisons of CSF cytokine/chemokine levels between untreated and treated patients.
