# Supplementary material for: Intrathecal cytokine profile in neuropathy with anti‐neurofascin 155 antibody
Source: Ann Clin Transl Neurol. 2019 Oct 27;6(11):2304–16. doi: 10.1002/acn3.50931 (PMC6856599; doi:10.1002/acn3.50931)
Supplement: Supplementary file 1 — Table S1 . Detection rates of cytokines/chemokines/growth factors in CSF. Table S2 . Comparisons of clinical and laboratory findings between anti‐NF155 antibody‐negative CIDP patients with high and low IL‐1β levels. Figure S1 . CSF protein amounts, cell counts, and cytokine/chemokine/growth factor levels in untreated patients. Figure S2 . Correlations of cytokine/chemokine levels with clinical severity and CSF protein amounts and cell counts in untreated patients. Figure S3 . Comparisons of CSF cytokine/chemokine levels between untreated and treated patients. [file ACN3-6-2304-s001.docx]

**Supplementary data**

**Intrathecal cytokine profile in neuropathy with anti-neurofascin 155 antibody**

Hidenori Ogata, MD, PhD,^1^* Xu Zhang, MD,^1,2^* Ryo Yamasaki, MD, PhD,^1^ Takayuki Fujii, MD, PhD,^1^ Akira Machida, MD, PhD,^3^ Nobutoshi Morimoto, MD, PhD,^4^ Kenichi Kaida, MD, PhD,^5^ Teruaki Masuda, MD, PhD,^6^ Yukio Ando, MD, PhD,^6^ Motoi Kuwahara, MD, PhD,^7^ Susumu Kusunoki, MD, PhD,^7^ Yuri Nakamura, MD, PhD,^1^ Takuya Matsushita, MD, PhD,^1^ Noriko Isobe, MD, PhD,^8^ and Jun-ichi Kira, MD, PhD^1^**

^1^Department of Neurology, Neurological Institute, Graduate School of Medical Sciences, Kyushu University, Fukuoka, Japan

^2^Department of Neurology and Tianjin Neurological Institute, Tianjin Medical University General Hospital, Tianjin, China

^3^Department of Neurology, Tsuchiura Kyodo General Hospital, Ibaraki, Japan

^4^Department of Neurology, Kagawa Prefectural Central Hospital, Kagawa, Japan

^5^Department of Neurology, Anti-aging and Vascular Medicine, National Defense Medical College, Saitama, Japan

^6^Department of Neurology, Graduate School of Medical Sciences, Kumamoto University, Kumamoto, Japan

^7^Department of Neurology, School of Medicine, Kinki University, Osaka, Japan

^8^Department of Neurological Therapeutics, Neurological Institute, Graduate School of Medical Sciences, Kyushu University, Fukuoka, Japan

*These authors contributed equally to this manuscript.

**Corresponding author.

**Supplementary Table 1. Detection rates of cytokines/chemokines/growth factors in CSF.**

| **Cytokines/ chemokines/ growth factors** | **Lower detection limit (pg/mL)** | **Upper detection limit (pg/mL)** | **Detection rate (%)** | | | | **Chi-square test** | **Fisher’s exact probability test (*p^corr^*)** | | |
| --- | --- | --- | --- | --- | --- | --- | --- | --- | --- | --- |
|  |  |  | **Total (n=100)** | **NF155^+^ CIDP (n=35)** | **NF155^−^ CIDP (n=36)** | **NIND (n=28)** | **(*p-*value)** | **NF155^+^ *vs.* NF155^−^** | **NF155^+^ *vs.* NIND** | **NF155^−^ *vs.* NIND** |
| **Pleiotropic cytokines** | | | | |  |  |  |  |  |  |
| IL-1β | 0.06 | 1019.37 | 73.0 | 42.9 | 80.5 | 96.4 | <0.0001 | 0.0033 | <0.0001 | NS |
| IL-6 | 0.49 | 7798.98 | 87.0 | 82.9 | 88.9 | 89.3 | NS |  |  |  |
| TNF-α | 3.21 | 47457.42 | 93.0 | 100 | 94.4 | 82.1 | 0.0208 | NS | 0.0276 | NS |
| IL-7 | 2.16 | 27222.36 | 37.0 | 34.3 | 38.9 | 35.7 | NS |  |  |  |
| **Th1-related cytokines** | | | | |  |  |  |  |  |  |
| IL-2 | 1.02 | 16994.31 | 5.0 | 2.9 | 5.6 | 7.1 | NS |  |  |  |
| IL-12 | 1.13 | 19351.08 | 25.0 | 22.9 | 27.8 | 25.0 | NS |  |  |  |
| IL-15 | 5.80 | 110339.72 | 7.0 | 2.7 | 13.9 | 3.6 | NS |  |  |  |
| IFN-γ | 0.27 | 4539.53 | 100 | 100 | 100 | 100 | NS |  |  |  |
| **Th2-related cytokines** | | | | |  |  |  |  |  |  |
| IL-4 | 0.08 | 1707.83 | 60.0 | 68.8 | 38.9 | 75.0 | 0.0057 | 0.0366 | NS | 0.0117 |
| IL-5 | 3.53 | 50761.57 | 67.0 | 71.4 | 61.1 | 67.9 | NS |  |  |  |
| IL-9 | 2.29 | 11659.44 | 50.0 | 37.1 | 52.8 | 60.7 | NS |  |  |  |
| IL-10 | 1.69 | 21424.56 | 100 | 100 | 100 | 100 | NS |  |  |  |
| IL-13 | 0.27 | 4687.25 | 98.0 | 100 | 86.1 | 100 | NS |  |  |  |
| **Th17-related cytokine** | | | | |  |  |  |  |  |  |
| IL-17 | 1.70 | 27205.2 | 68.0 | 71.4 | 66.7 | 64.3 | NS |  |  |  |
| **Tfh-related cytokine** | | | | |  |  |  |  |  |  |
| IL-21 | 28.17 | 103802.12 | 2.0 | 0 | 2.8 | 3.6 | NS |  |  |  |
| **Chemokines** |  |  |  |  |  |  |  |  |  |  |
| CXCL8/IL-8 | 1.04 | 18188.08 | 100 | 100 | 100 | 100 | NS |  |  |  |
| CCL11/Eotaxin | 0.12 | 2071.72 | 100 | 100 | 100 | 100 | NS |  |  |  |
| CXCL10/IP-10 | 3.52 | 13960.66 | 100 | 100 | 100 | 100 | NS |  |  |  |
| CCL2/MCP-1 | 0.34 | 1917.88 | 100 | 100 | 100 | 100 | NS |  |  |  |
| CCL3/MIP-1α | 0.04 | 597.38 | 100 | 100 | 94.4 | 100 | NS |  |  |  |
| CCL4/MIP-1β | 0.40 | 7441.85 | 100 | 100 | 100 | 100 | NS |  |  |  |
| CCL5/RANTES | 3.66 | 3710.42 | 100 | 100 | 100 | 100 | NS |  |  |  |
| **Growth factors** | | | | |  |  |  |  |  |  |
| G-CSF | 5.80 | 97238.11 | 93.0 | 88.6 | 91.7 | 100 | NS |  |  |  |
| GM-CSF | 0.40 | 5577.81 | 10.0 | 2.9 | 16.7 | 10.7 | NS |  |  |  |
| PDGF-BB | 4.21 | 19182.91 | 31.0 | 45.7 | 30.6 | 14.3 | 0.0275 | NS | 0.0399 | NS |
| bFGF | 3.47 | 3855.17 | 22.0 | 5.7 | 25.0 | 35.7 | 0.0119 | NS | 0.0078 | NS |
| VEGF | 13.33 | 120683.14 | 4.0 | 0 | 11.1 | 0 | NS |  |  |  |
| **Anti-inflammatory cytokine** | | | | |  |  |  |  |  |  |
| IL-1ra | 4.21 | 19230.98 | 76.0 | 60.0 | 72.2 | 100 | 0.0009 | NS | <0.0001 | 0.0102 |

No samples had levels beyond the upper detection limits. Detection rates are expressed as percentages of samples above the lower detection limit in each group. Significant differences in detection rates between each set of two groups are shown as corrected *p-*values (*p^corr^*; *p-*values from Fisher’s exact probability test were corrected by Bonferroni–Dunn’s correction). Detection rates of TNF-α and PDGF-BB were higher in NF155^+^ CIDP patients than in NIND patients (*p* = 0.0276 and *p* = 0.0399, respectively), while the detection rate of bFGF was lower in NF155^+^ CIDP patients than in NIND patients (*p* = 0.0078). The detection rate of IL-1β was lower in NF155^+^ CIDP patients than in NF155^−^ CIDP and NIND patients (*p* = 0.0033 and *p* < 0.0001, respectively). The detection rate of IL-1ra was lower in both NF155^+^ CIDP and NF155^−^ CIDP patients than in NIND patients (*p* < 0.0001 and *p* = 0.0102, respectively). The detection rate of IL-4 was lower in NF155^−^ CIDP patients than in NF155^+^ CIDP and NIND patients (*p* = 0.0366 and *p* = 0.0117, respectively). The detection rates of other cytokines/chemokines/growth factors showed no significant differences between NF155^+^ CIDP, NF155^−^ CIDP, and NIND patients. IL-2, IL-12, IL-15, IL-21, bFGF, GM-CSF, and VEGF were excluded from further statistical analysis because of their low detection rates. bFGF = basic fibroblast growth factor; CCL = C-C motif ligand; CIDP = chronic inflammatory demyelinating polyneuropathy; CSF = cerebrospinal fluid; CXCL = C-X-C motif ligand; G-CSF = granulocyte colony-stimulating factor; GM-CSF = granulocyte-macrophage colony-stimulating factor; IFN = interferon; IL = interleukin; IL-1ra = interleukin-1 receptor antagonist; IP-10 = interferon-γ gamma-inducible protein-10; MCP-1 = monocyte chemoattractant protein-1; MIP = macrophage inflammatory protein; NF155 = neurofascin 155; NF155^+^ = IgG4 anti-NF155 antibody-positive ; NF155^−^ = anti-NF155 antibody-negative; NIND = non-inflammatory neurological disease; NS = not significant; PDGF-BB = platelet-derived growth factor; RANTES = regulated upon activation, normal T cell expressed and secreted; Tfh = follicular helper T; Th1 = type 1 T helper; Th2 = type 2 T helper; Th17 = type 17 T helper; TNF = tumor necrosis factor; VEGF = vascular endothelial growth factor.

**Supplementary Table 2. Comparisons of clinical and laboratory findings between anti-NF155 antibody-negative CIDP patients with high and low IL-1β levels.**

|  | NF155^-^ CIDP with low IL-1β* (N = 18) | NF155^-^ CIDP with high IL-1β* (N = 18) | *p* value** |
| --- | --- | --- | --- |
| Female:Male (%) | 1:1.6 (38.9) | 1:3.5 (22.2) | NS |
| Age at sample collection, median (range), y | 53 (11-77) | 57.5 (21-81) | NS |
| Age at onset, median (range), y | 46 (10-76) | 48 (15-71) | NS |
| CSF total proteins, mean (SD), mg/dl | 134.1 (85.2) | 49.7 (19.6) | <0.0001 |
| CSF cell counts, mean (SD), /ul | 1.8 (1.2) | 2.4 (2.9) | NS |
| Clinical phenotype | n/N (%) | n/N (%) |  |
| Typical | 16/18 (88.9) | 8/18 (44.4) | 0.0063 |
| DADS | 0/18 (0) | 2/18 (11.1) | NS |
| MADSAM | 1/18 (5.6) | 4/18 (22.2) | NS |
| Others | 1/18 (5.6) | 4/18 (22.2) | NS |

*NF155^−^ CIDP patients were divided into two groups using the median value of IL-1β (1.06 mg/dl).

***p* < 0.05 = significant difference. CIDP = chronic inflammatory demyelinating polyneuropathy; CSF = cerebrospinal fluid; DADS = distal acquired demyelinating symmetric neuropathy; MADSAM = multifocal acquired demyelinating sensory and motor neuropathy; NF155 = neurofascin 155; NF155^−^ = anti-NF155 antibody-negative; NA = not applicable; NS = not significant; SD = standard deviation; y = year.


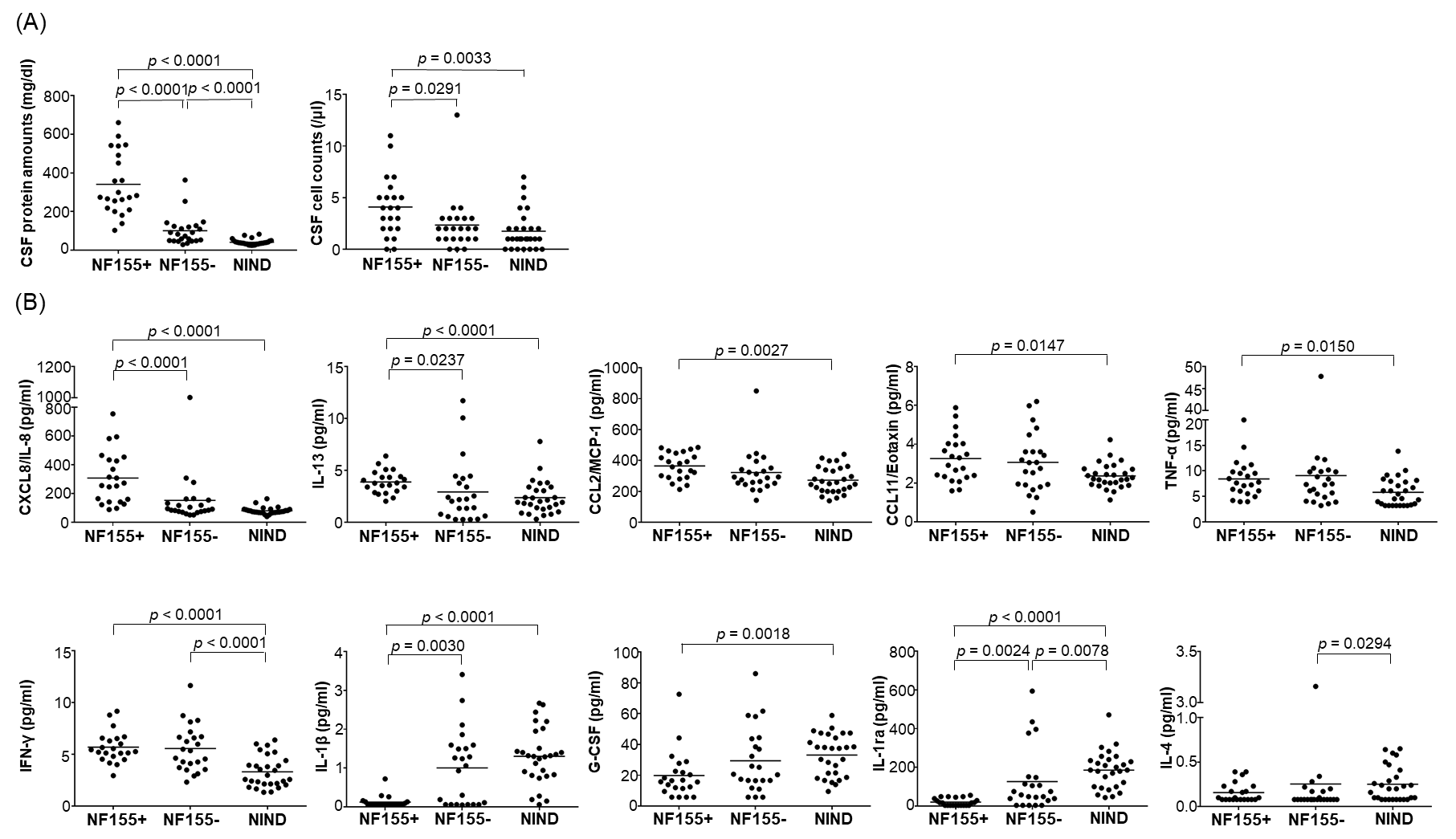
 **Supplementary Figure 1. CSF protein amounts, cell counts, and cytokine/chemokine/growth factor levels in untreated patients.**

(A, B) CSF protein amounts and cell counts (A) and levels of CSF cytokines/chemokines and growth factors (B) showing significant differences between untreated IgG4 anti-neurofascin 155 (NF155) antibody-positive CIDP (NF155^+^), untreated anti-NF155 antibody-negative CIDP (NF155^−^), and non-inflammatory neurological disease (NIND) patients. CCL = C-C motif ligand; CIDP = chronic inflammatory demyelinating polyneuropathy; CXCL = C-X-C motif ligand; G-CSF = granulocyte colony-stimulating factor; IFN = interferon; IL = interleukin; IL-1ra = interleukin-1 receptor antagonist; MCP-1 = monocyte chemoattractant protein-1; TNF-α = tumor necrosis factor-α.

**
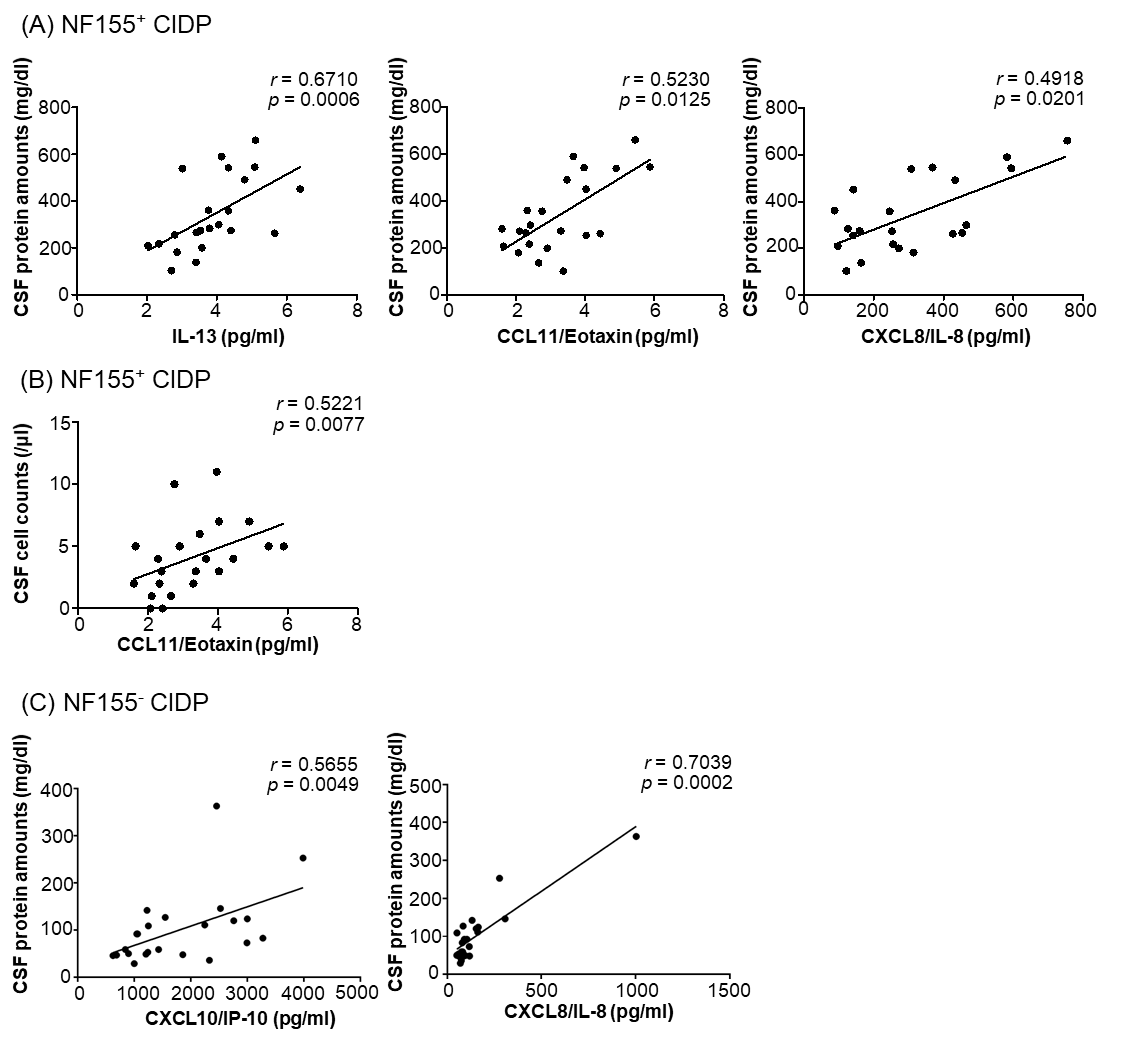
Supplementary Figure 2. Correlations of cytokine/chemokine levels with clinical severity and CSF protein amounts and cell counts in untreated patients.**

(A, B) CSF cytokines and chemokines showing positive correlations with CSF protein amounts (A) and cell counts (B) in untreated patients with IgG4 anti-neurofascin 155 (NF155) antibody-positive (NF155^+^) CIDP. (C) CSF cytokines and chemokines showing positive correlations with CSF protein amounts in untreated patients with anti-NF155 antibody-negative (NF155^−^) CIDP. Only pretreatment samples were evaluated and Spearman’s rank correlation coefficient was used for this analysis. CCL = C-C motif ligand; CIDP = chronic inflammatory demyelinating polyneuropathy; CXCL = C-X-C motif ligand; G-CSF = granulocyte colony-stimulating factor; IFN = interferon; IL = interleukin; IL-1ra = interleukin-1 receptor antagonist; IP-10 = interferon-γ gamma-inducible protein-10.

**Supplementary Figure 3. Comparisons of CSF cytokine/chemokine levels between untreated and treated patients.**

**
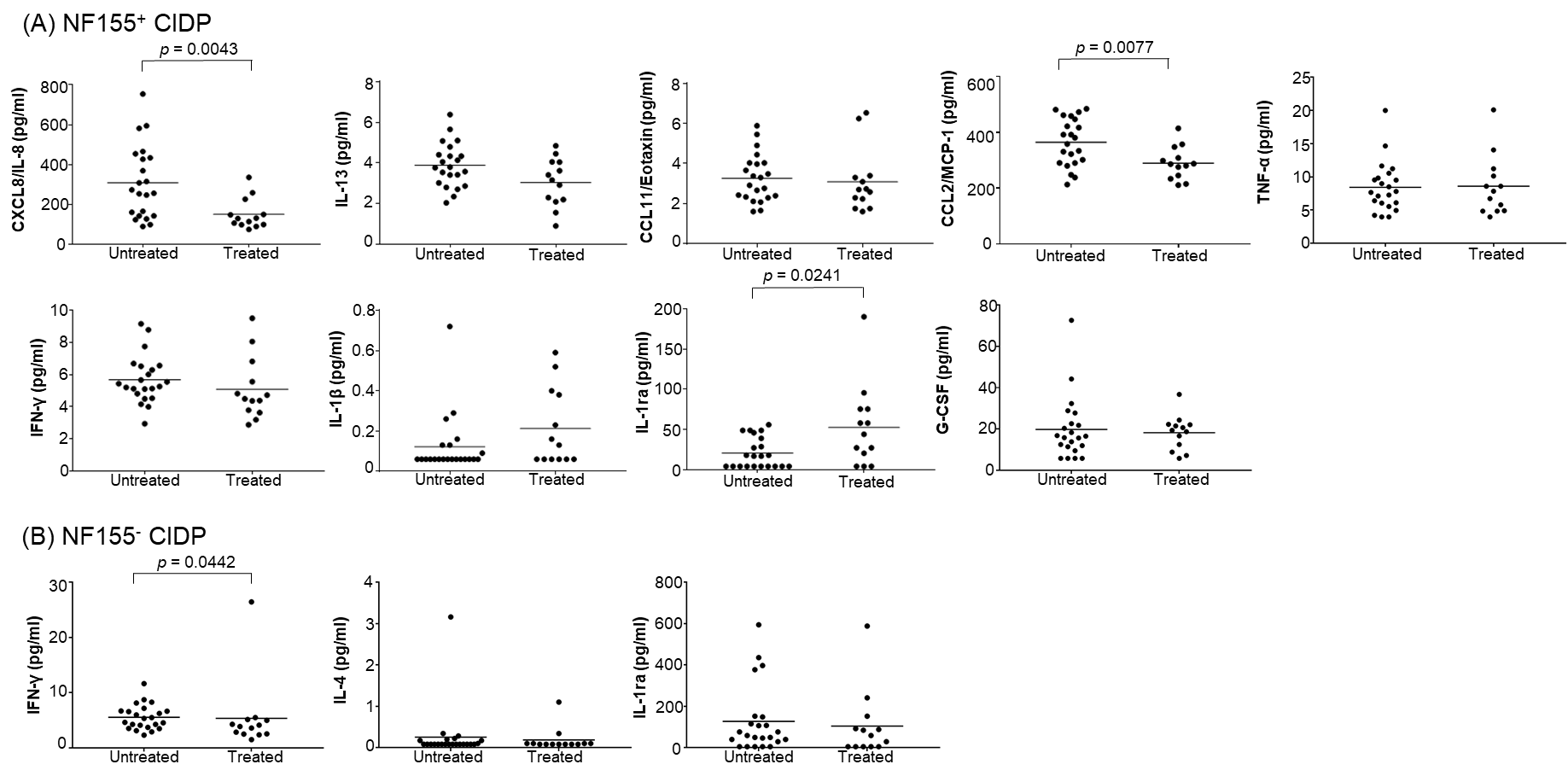
**

(A, B) CSF cytokine/chemokine levels showing significant differences from those in non-inflammatory neurological disease patients were compared between untreated and treated samples from IgG4 anti-NF155 antibody-positive (NF155^+^) CIDP (A) and anti-NF155 antibody-negative (NF155^−^) CIDP (B) patients. CIDP = chronic inflammatory demyelinating polyneuropathy; CSF = cerebrospinal fluid; CCL= C-C motif ligand; CXCL = C-X-C motif ligand; G-CSF = granulocyte colony-stimulating factor; IFN = interferon; IL = interleukin; IP-10 = interferon-γ-inducible protein-10; MCP-1 = monocyte chemoattractant protein-1; MIP = macrophage inflammatory protein; PDGF = platelet-derived growth factor; RANTES = regulated upon activation, normal T cell expressed and secreted; TNF = tumor necrosis factor.
